# One Week of Single-Leg Immobilization Lowers Muscle Connective Protein Synthesis Rates in Healthy, Young Adults

**DOI:** 10.1249/MSS.0000000000003342

**Published:** 2023-11-22

**Authors:** ANDREW M. HOLWERDA, MICHELLE E. G. WEIJZEN, ANTOINE ZORENC, JOAN SENDEN, GUUS H. J. JETTEN, LISANNE H. P. HOUBEN, LEX B. VERDIJK, LUC J. C. VAN LOON

**Affiliations:** Department of Human Biology, School of Nutrition and Translational Research in Metabolism (NUTRIM), Maastricht University Medical Centre+, Maastricht, THE NETHERLANDS

**Keywords:** COLLAGEN, INJURY, DISUSE, EXTRACELLULAR MATRIX, REHABILITATION

## Abstract

**Purpose:**

Short periods of limb immobilization lower myofibrillar protein synthesis rates. Within skeletal muscle, the extracellular matrix of connective proteins is recognized as an important factor determining the capacity to transmit contractile force. Little is known regarding the impact of immobilization and subsequent recovery on muscle connective protein synthesis rates. This study examined the impact of 1 wk of leg immobilization and 2 wk of subsequent ambulant recovery on daily muscle connective protein synthesis rates.

**Methods:**

Thirty healthy, young (24 ± 5 yr) men were subjected to 7 d of one-legged knee immobilization followed by 14 d of ambulant recovery. Deuterium oxide ingestion was applied over the entire period, and muscle biopsy samples were collected before immobilization, after immobilization, and after recovery to measure muscle connective protein synthesis rates and mRNA expression of key extracellular matrix proteins (collagen I, collagen III), glycoproteins (fibronectin, tenascin-C), and proteoglycans (fibromodulin, and decorin). A two-way repeated-measures (time–leg) ANOVA was used to compare changes in muscle connective protein synthesis rates during immobilization and recovery.

**Results:**

During immobilization, muscle connective protein synthesis rates were lower in the immobilized (1.07 ± 0.30%·d^−1^) compared with the nonimmobilized (1.48 ± 0.44%·d^−1^; *P* < 0.01) leg. When compared with the immobilization period, connective protein synthesis rates in the immobilized leg increased during subsequent recovery (1.48 ± 0.64%·d^−1^; *P* < 0.01). After recovery, skeletal muscle collagen I, collagen III, fibronectin, fibromodulin, and decorin mRNA expression increased when compared with the postimmobilization time point (all *P* < 0.001).

**Conclusions:**

One week of leg immobilization lowers muscle connective protein synthesis rates. Muscle connective protein synthesis rates increase during subsequent ambulant recovery, which is accompanied by increased mRNA expression of key extracellular matrix proteins.

Recovery from injury or illness often requires a period of inactivity. Periods of inactivity strongly reduce muscle mass and strength (1,2), which can lead to impaired metabolic health and a decline in functional capacity (3). The reduction in muscle mass has been primarily attributed to a decline in muscle protein synthesis rates (4–6), which results in a negative net muscle protein balance. When compared with the loss of muscle mass, the decline in muscle strength has been shown to occur up to fivefold more rapidly (1,2,7), indicating that factors other than muscle mass loss *per se* contribute to strength loss observed after a period of disuse.

Within skeletal muscle, the extracellular matrix is recognized as an important factor determining the capacity to transmit contractile force (8,9). For instance, the extracellular matrix has been shown to transmit most (~80%) of contractile force from adjacent muscle fibers to the tendon to induce joint movement (10,11). Consequently, changes in extracellular matrix quality (e.g., increased/decreased stiffness) can have an impact on the contractile and force-transferring abilities of skeletal muscle tissue (10,12,13). Despite the importance of the extracellular matrix for contractile force transfer, the impact of changes in physical activity on connective protein remodeling remains far from understood. Several studies have demonstrated that increased mechanical loading (i.e., resistance-type exercise) potently upregulates muscle connective protein synthesis rates (14–18). Furthermore, greater loading induced by lengthening contractions has been shown to increase muscle connective protein synthesis rates when compared with shortening contractions (19). Although the anabolic impact of increased mechanical loading has been well established (14–18), no study has evaluated the impact of immobilization on muscle connective protein synthesis rates in humans. However, earlier rodent work has demonstrated that short-term limb immobilization potently downregulates mRNA expression of connective proteins (i.e., collagens I and III) (20,21). Here, we hypothesize that short-term immobilization lowers daily muscle connective protein synthesis rates.

The responsiveness of muscle protein synthesis to remobilization forms an important factor in determining the recovery process after limb immobilization. Despite its importance, we are aware of only two studies have assessed the muscle protein synthetic response following the return to regular physical activity after short-term immobilization (22). Mitchell et al. (22) failed to detect a decline in myofibrillar protein synthesis rates during 14 d of immobilization and reported an increase in myofibrillar protein synthesis rates during 14 d of subsequent remobilization only when additional protein was supplemented on top of a dietary protein intake of 1 g·kg^−1^·d^−1^. Weijzen et al. (2) recently showed a decline in myofibrillar protein synthesis rates during 7 d of immobilization, which was increased during 14 d of ambulant recovery. To date, however, no study has evaluated the muscle connective protein synthetic response during recovery from short-term limb immobilization. Here, we hypothesized that remobilization after a short period of disuse increases muscle connective protein synthesis rates.

To test our hypotheses, we recruited 30 healthy young men to be subjected to 1 wk of single-leg immobilization, followed by a 2-wk period of remobilization. Muscle connective protein synthesis rates were assessed during short-term immobilization and subsequent 2 wk of recovery. Before and immediately after immobilization and after 2 wk of subsequent recovery, plasma collagen turnover markers, muscle collagen content, and mRNA expression of extracellular matrix proteins were assessed.

## METHODS

### Participants

Thirty healthy, young men (24 ± 5 yr) were included in the present study. Exclusion criteria included the following: (family) history of thrombosis; (family) history of Factor V Leiden or other known thrombophilia (such as; protein C, protein S, antithrombin deficiency); lower limb; back or shoulder injuries that could interfere with the use of crutches; allergies to milk protein; participation in a structured resistance exercise program; comorbidities interacting with mobility and muscle metabolism of the lower limbs (e.g., arthritis, spasticity/rigidity, all neurological disorders and paralysis); use of any medications known to (or that may) affect protein metabolism; diagnosed diabetes; metabolic, cardiovascular, or intestinal disorders; a history of neuromuscular problems; use of anticoagulants; use of protein and/or fish-oil supplements; participation in a ^2^H_2_O study in the previous 6 months; and smoking. On the screening visit, all participants were fully informed about the nature and risks of the experimental procedures before providing informed consent. In addition to screening for thrombosis risk factors, the risk of thrombosis formation during immobilization was minimized by collecting the baseline biopsy from the nonimmobilized leg and instructing the subjects to perform non–weight-bearing exercises (foot flexion/extension, foot rotations, hip flexion) three times per day during the immobilization period. Participants were monitored closely during the entire study for complaints (i.e., red, warm, or swollen skin). This study was approved by the local Medical Ethical Committee of Maastricht University Medical Centre+ and conforms to the principles outlined in the latest version of the Declaration of Helsinki for use of human subjects and tissue. This trial was registered at https://trialsearch.who.int/ as NL7645. The study was independently monitored by the Clinical Trial Center Maastricht. This study is part of a greater project comparing the impact of milk protein versus nutritional peptide supplementation on muscle mass, strength, and myofibrillar protein synthesis rates during 7 d of limb immobilization and 14 d of subsequent recovery (2). The results presented herein are novel and do not overlap with the greater project.

### Experimental design

**FIGURE 1:**
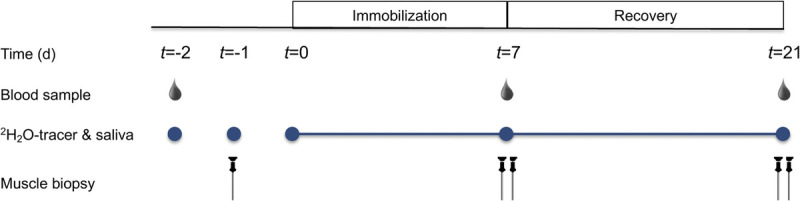
Schematic representation of experimental protocol.

A schematic overview of the experimental design is depicted in Figure 1. In the original study, participants were randomly allocated to supplement isonitrogenous amounts of either a *Vicia faba–*derived peptide network (NPN_1; *n* = 15) or milk protein concentrate (MPC; *n* = 15) (2). All participants were subjected to 7 d of knee immobilization on a randomized leg followed by 14 d of free-living (ambulant) recovery. Participants underwent a deuterated water (^2^H_2_O) dosing protocol, which started 2 d before casting and continued until the end of the experimental trial. Muscle biopsies from both the immobilized and nonimmobilized legs and venous blood samples were taken before casting, after casting, and after the recovery period.

### Leg immobilization and habitual recovery

Each participant was fitted for a full-leg plaster cast in a randomized and counterbalanced (for leg) fashion to induce knee immobilization. The cast extended from 10 cm above the ankle to 25 cm above the patella and was set at a 30° flexion angle. Throughout the immobilization period, participants were provided with crutches to allow movement, but were instructed to not put any weight on the immobilized leg. Participants were instructed to perform a series of ankle exercises (i.e., plantar and dorsal flexion, and circular foot movements) to avoid developing deep vein thrombosis. After cast removal, participants were transported by wheelchair until the muscle biopsy sample was collected. After the postimmobilization visit (*t* = 7 d), the 14-d recovery period started.

### Dietary intake and physical activity

Participants were instructed to refrain from strenuous physical activity, avoid alcohol intake, and keep their diet as constant as possible for 2 d before the first experimental test day until the final test visit (*t* = 21 d). All participants received a standardized meal before test days on *t* = −1, 7, and 21 d (2.9 MJ, 53 En% carbohydrate, 31 En% fat, 16 En% protein). Dietary intake and physical activity records were completed by the participants for 3 d before the immobilization period and during the final 3 d of the immobilization and recovery periods. Dietary intake records were analyzed using the Dutch Food Consumption Database 2019 (NEVO; RIVM, Bilthoven, the Netherlands) (23). Daily steps were recorded over the same 3-d periods as the dietary intake and physical activity diaries using a triaxial accelerometer (Actigraph GT3X; Actigraph LLC, Pensacola, FL), worn on the waist. Data were included in the analysis if participants wore the Actigraph for a minimum of 2 d and at least 10 h per day.

### Body composition

Body weight was measured with a digital balance with an accuracy of 0.1 kg (SECA GmbH, Hamburg, Germany). Body composition (fat, fat-free mass, and bone mineral content) was determined by DXA scan (Hologic Discovery A, Marlborough, MA). The system’s software package APEX version 4.0.2 was used to determine whole body and regional (e.g., legs) lean mass, fat mass, and bone mineral content.

### Muscle strength

Single-legged leg extension one-repetition maximum (1RM) was determined using the successive attempts whereby the load was increased after each successful lift until failure was reached (2). Three-minute rest periods were allowed between the lifts. A repetition was considered valid when the participant was able to complete the entire lift in a controlled manner without assistance.

### Deuterated water-dosing protocol

The deuterated water-dosing protocol consisted of 1 dosing day and 22 maintenance days. The dosing protocol was modified from previously published studies that have administered deuterated water in human participants (24–26). In the morning in an overnight fasted state, baseline blood and saliva samples were collected. Participants then ingested two doses of 100 mL of 70% deuterium oxide (^2^H_2_O; Cambridge Isotopes Laboratories, Andover, MA) separated by 30–60 min. Participants ingested 20 mL of 70% deuterium oxide every morning for the remainder of the trial to maintain deuterium enrichment in the body water and alanine pools.

### Saliva and plasma sampling

To assess body water enrichment (^2^H_2_O), participants collected saliva samples using a dental swab (Celluron, Hartmann, Germany) every evening for the entire experimental protocol. Participants were instructed to not eat or drink anything 30 min before saliva collection.

Blood samples were collected at *t* = −2, −1, 0, 7, and 21 d in EDTA-containing tubes and centrifuged at 1000*g* for 10 min at 4°C. Aliquots of plasma were frozen in liquid nitrogen and stored at −80°C. Before the immobilization period, a muscle biopsy was collected from the vastus lateralis muscle of the leg identified as the leg that would not be immobilized (nonimmobilized leg).

### Muscle tissue sampling

On the visits after immobilization and recovery, muscle biopsies from both the immobilized and the nonimmobilized leg were collected. Muscle biopsy samples were obtained from the middle region of the vastus lateralis, approximately 1–3 cm below the level where the computed tomography (CT) scan was performed, by using the percutaneous needle biopsy technique (27). Muscle samples were dissected carefully, freed from any visible nonmuscle material, frozen in liquid nitrogen, and stored at −80°C until further analyses.

### Plasma analyses

Procollagen type I N propeptide (PINP) and carboxy-terminal crosslinking telopeptide of type I collagen (CTX-I) were selected as markers of bone/collagen formation and bone/collagen resorption, respectively, in line with the recommendation by The International Osteoporosis Foundation and International Federation of Clinical Chemistry (28). PINP and CTX-I were measured at *t* = −2, 0, 7, and 21 d. Intact PINP and CTX-I were measured using chemiluminescent immunometric assays on the IDS-iSYS instrument (Immunodiagnostic Systems, PLC), by the Central Diagnostic Laboratory at the Maastricht University Medical Centre (Maastricht, the Netherlands).

Plasma free [^2^H]alanine enrichments were determined by gas chromatography–mass spectrometry analysis (Agilent 5975C MSD & 7890A GC, Wilmington, DE) on *t* = −2, −1, 0, 7, and 21 d, as described previously (25).

### Muscle collagen content

Collagen content in the muscle biopsy samples was determined using the collagen-specific amino acid, 4-hydroxyproline. Tissue 4-hydroxyproline concentrations were performed using ultraperformance liquid chromatograph mass spectrometry (ACQUITY UPLC H-Class with QDa; Waters, Saint-Quentin, France), as described in detail previously (29). To determine collagen content, tissue 4-hydroxyproline concentrations were multiplied by 7.5 and expressed relative to dry tissue weight (30).

### Muscle connective protein synthesis rates

Connective protein-enriched fractions were extracted from ~70 mg wet muscle tissue by hand-homogenizing on ice using a pestle in a standard extraction buffer (10 μL·mg^−1^). The samples were centrifuged at 700*g* and 4°C for 15 min. The pellet was washed with 400 μL extraction buffer and centrifuged at 700*g* and 4°C for 10 min. Supernatant was removed, and the pellet was washed with 500 μL milliQ water before vortexing and centrifugation at 700*g* and 4°C for 10 min. Supernatant was removed and 1 mL of homogenization buffer was added, and the material was suspended by vortexing before transferring into microtubes containing 1.4-mm ceramic beads and Lysing Matrix D (MP Biomedicals, Irvine, CA). The samples were shaken four times for 45 s at 5.5 m·s^−1^ (FastPrep-24 5G; MP Biomedicals) to mechanically lyse the protein network. Samples were left to rest at 4°C for 3 h before centrifuging at 800*g* and 4°C for 20 min. Supernatant was discarded, and 1 mL of homogenization buffer was added. The microtubes were shaken once for 45 s and 5.5 m·s^−1^ and left to rest at 4°C for 30 min before centrifuging at 800*g* and 4°C for 20 min. Supernatant was discarded and 1 mL KCl buffer was added to the pellet, and samples were left to rest overnight at 4°C. The next morning, samples were vortexed, transferred to new microtubes, and centrifuged at 1600*g* and 4°C for 20 min. The pellet containing immature and mature connective proteins was collected. The pellet was mixed with 1 mL KCl buffer and left for 2 h at 4°C. The samples were vortexed and centrifuged at 1600*g* for 20 min at 4°C, and the supernatant was discarded. To the pellet, 1 mL ddH_2_O was added, vortexed, left for 2 h at 4°C, and then centrifuged at 1600*g* for 20 min at 4°C. The supernatant was removed and the remaining pellet was washed once with 100% ethanol, once with 70% ethanol and hydrolyzed overnight in 2 mL of 6 M HCl at 110°C.

The free amino acids from the hydrolyzed connective protein pellet were dried under a continuous nitrogen stream while being heated at 120°C. The free amino acids were then dissolved in 25% acetic acid solution, passed over cation exchange AG 50 W-X8 resin columns (mesh size: 100–200, ionic form: hydrogen; Bio-Rad Laboratories, Hercules, CA), and eluted with 2 M NH_4_OH. Thereafter, the eluate was dried, and the purified amino acids were derivatized to their N(O,S)-ethoxycarbonyl ethyl esters. The derivatized samples were measured using a gas chromatography–isotope ratio mass spectrometer (MAT 253; Thermo Fisher Scientific, Bremen, Germany) equipped with a pyrolysis oven and a 60-m DB-17MS column (no. 122-4762; Agilent, Wilmington, DE) and 5-m precolumn. Ion masses 2 and 3 were monitored to determine the ^2^H/^1^H ratios of muscle protein-bound alanine. A series of known standards were applied to assess linearity of the mass spectrometer and to control for the loss of tracer.

### Reverse transcription polymerase chain reaction

Total RNA was isolated and quantified from 10 to 20 mg of frozen muscle tissue using TRIzol^®^ Reagent (Life Technologies, Invitrogen, Bleiswijk, the Netherlands), according to the manufacturer’s protocol, as described previously (7). Taqman primer/probe sets were obtained from Fisher Emergo: collagen I (Hs00164004_m1), collagen III (Hs00943809_m1), fibronectin (Hs01549976_m1), tenascin-C (Hs01115665_m1), fibromodulin (Hs00157619_m1), decorin (Hs00370385_m1), and 18S (Hs03003631_g1). The housekeeping gene 18S was used as an internal control, as this gene was unaffected by immobilization (mean CT values were unaffected over time; data not shown) and has been used previously in similar studies (7,31). Relative quantification of the genes was performed using the ΔΔCT method (2^–ΔΔCt^). Ct values of the target genes were normalized to Ct values of the internal control, and results were calculated as relative expression against the standard curve. The Ct values of all genes of interest were always within the lower and upper boundaries of the standard curve.

### Calculations

Connective protein fractional synthetic rate (FSR) was determined using the incorporation of [^2^H]alanine into muscle proteins and the mean plasma [^2^H]alanine enrichment as a precursor. As we assessed FSR for >14 d, the nonlinear equation was used to calculate FSR as described earlier (2,32):


$$\mathrm{FSR}\;\left(\%/d\right)=\frac{-\ln\;\left(1-f\right)}{t}\times 100$$

$$f=\frac{E_{m2}-E_{m1}}{E_{\text{precursor}}}$$

where *f* is calculated as the change in connective protein-bound [^2^H]alanine enrichment between consecutive biopsy samples (*E*_m2_ − *E*_m1_) divided by the mean precursor [^2^H]alanine enrichment (*E*_precursor_), and *t* represents the time between consecutive biopsy samples on days −1 and *7*, or between days 7 and 21.

### Statistics

The primary outcome variable was muscle connective protein synthesis rates. In the original study (2), participants were randomly selected to receive either MPC or peptide supplementation throughout the immobilization and recovery periods. A power calculation was performed for the original study (2), resulting in 30 subjects required to detect a 40% difference in the change in quadriceps cross-sectional area between the nutritional interventions over the 7-d immobilization period. Based on the original study (2) and our previous studies reporting muscle connective protein synthesis rates (14,15), we expected that *n* = 29 would be sufficient to detect at least a 20% difference in muscle connective protein synthesis rates between the immobilized and nonimmobilized leg during the immobilization period (considering an SD of 0.3%·d^−1^, *α* of 0.05, and 1 − *β* of 0.8). Data for the primary outcome were normally distributed, whereas part of the data for the secondary outcomes did not follow a normal distribution. However, because of the robustness of ANOVA to deviations from normality when sample sizes are equal and above *n* = 25, an ANOVA was conducted for all outcomes. In case of nonsphericity, the Greenhouse–Geisser correction was used. Data are expressed as mean ± SD, unless stated otherwise. Two-way repeated-measures ANOVAs during the immobilization period (leg–group) and over the entire experimental period (time–group) revealed no group or interaction effects for muscle connective protein synthesis rates as well as plasma collagen turnover markers, muscle collagen, and muscle mRNA expression (all *P* > 0.05). As such, all subsequent analyses were performed with all subjects in one group. A two-way repeated-measures ANOVA with leg (nonimmobilized vs immobilized) and time (immobilization vs recovery) as within-subject factors was applied to compare changes in muscle connective protein synthesis rates. A one-way ANOVA with time (preimmobilization, postimmobilization, and postrecovery) as the independent variable was applied to assess potential changes in plasma collagen turnover markers, muscle collagen, and muscle mRNA expression in only the immobilized leg over the course of the experiment. In case of significant interactions, Bonferroni *post hoc* tests were applied to locate differences. A Pearson’s correlation was performed to determine whether baseline daily step count was associated with the change in connective protein synthesis rates. Data were analyzed using SPSS version 27 (SPSS, IBM Corp., Armonk, NY). Statistical significance was set at *P* < 0.05.

## RESULTS

### Participants

Baseline participants’ characteristics are presented in (Table 1). Of the 30 participants, samples from 29 subjects were included in the present study, with 1 subject being excluded because of dropout during the COVID-19 lockdown.

**TABLE 1:** Participants’ characteristics.

	(n = 29)
Age, yr	24 ± 5
Height, m	1.77 ± 0.08
Body weight, kg	73.9 ± 11.8
BMI, kg·m^−2^	23.4 ± 2.5
Total lean mass, kg	54.0 ± 7.6
Leg (nonimmobilized) lean mass, kg	9.2 ± 1.4
Leg (immobilized) lean mass, kg	9.2 ± 1.2
Whole thigh (nonimmobilized) CSA, cm^2^	144 ± 20
Whole thigh (immobilized) CSA, cm^2^	145 ± 20
Fat, %	23.8 ± 5.5
Fasted glucose, mmol·L^−1^	4.9 ± 0.4
Systolic blood pressure, mm Hg	123 ± 11
Diastolic blood pressure, mm Hg	69 ± 10
Resting heart rate, bpm	65 ± 8

CSA, cross-sectional area.

### Dietary intake and physical activity

Dietary intake (including supplementation) and step count data are displayed in Table 2. Data of nine participants were excluded because of insufficient accelerometer wear time. No differences over time were observed for total energy intake, energy percentage of fat, energy percentage of carbohydrate, and total protein intake (all *P* > 0.05). During recovery, protein intake relative to body mass (*P* = 0.03) and energy percentage of protein (*P* = 0.048) were lower when compared with preimmobilization.

**TABLE 2:** Dietary intake and physical activity.

	Preimmobilization	Immobilization Period	Recovery Period
Nutrition*^a^*			
Energy intake, MJ·d^−1^	10.1 ± 2.8	9.2 ± 2.1	9.0 ± 2.0
Carbohydrate, En%	44 ± 8	48 ± 8	51 ± 9
Fat, En%	35 ± 6	38 ± 8	36 ± 9
Protein, En%	17 ± 4	18 ± 4	19 ± 4*
Protein intake, g·d^−1^	99 ± 28	97 ± 30	96 ± 22
Protein intake (g·kg^−1^·d^−1^	1.4 ± 0.4	1.3 ± 0.5	1.3 ± 0.4*
Physical activity*^b^*			
Step count, steps per day	6342 ± 1957	2203 ± 1299*	5547 ± 2451**

Data are displayed as means ± SD.

*^a^*Nutritional data include intake of the MPC and peptide supplements (2). Dietary intake data represent average values over 3 d before immobilization, and the final 3 d during the immobilization and recovery periods.

*^b^*Physical activity data are displayed for *n* = 17 because of insufficient accelerometer wearing time. Step count values represent average steps per day over 3 d before immobilization, and the final 3 d during the immobilization and recovery periods.

*Significantly different from the preimmobilization value (*P* < 0.05).

**Significantly different from the immobilization period value (*P* < 0.05).

During the final 3 d of immobilization, average daily step count was reduced to 2203 ± 1299 steps per day when compared with 6342 ± 1957 steps per day before immobilization (*P* < 0.001). During the final 3 d of recovery, average daily step count increased and was not different in comparison to preimmobilization values (*P* = 0.70).

### Muscle strength

Leg immobilization significantly decreased leg extension 1RM (56.7 ± 10.1 to 49.5 ± 9.9 kg, *P* < 0.001). After recovery, leg extension 1RM was greater compared with postimmobilization (52.9 ± 11.8 kg *P* < 0.008) but remained lower compared with before immobilization (*P* < 0.002).

### Plasma collagen turnover markers

**FIGURE 2:**
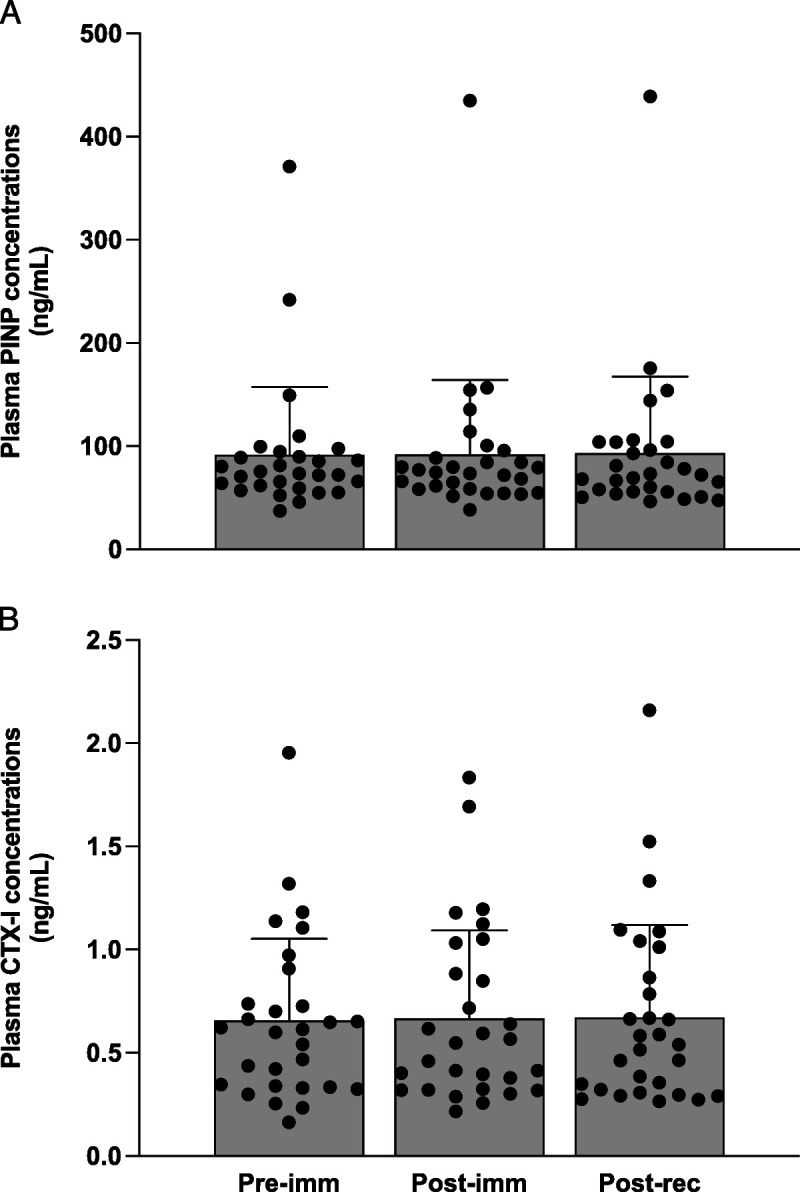
Plasma PINP (A; in nanograms per milliliter) and CTX-I (B; in nanograms per milliliter) concentrations at baseline, after 7 d of leg immobilization, and after 14 d of ambulatory recovery. The bars represent means ± SD (*n* = 29), and the dots represent individual values. Data were analyzed using a one-way ANOVA. No significant differences over time were observed in PINP (*F*_1.5, 43.3_ = 0.1, *P* = 0.85) or CTX-I (*F*_2, 56_ = 0.1, *P* = 0.92).

Plasma PINP concentrations did not change over time (Fig. 2A, *P* = 0.85). Plasma CTX-I concentrations did not change over time (Fig. 2B, *P* = 0.92).

### Muscle collagen contents

**FIGURE 3:**
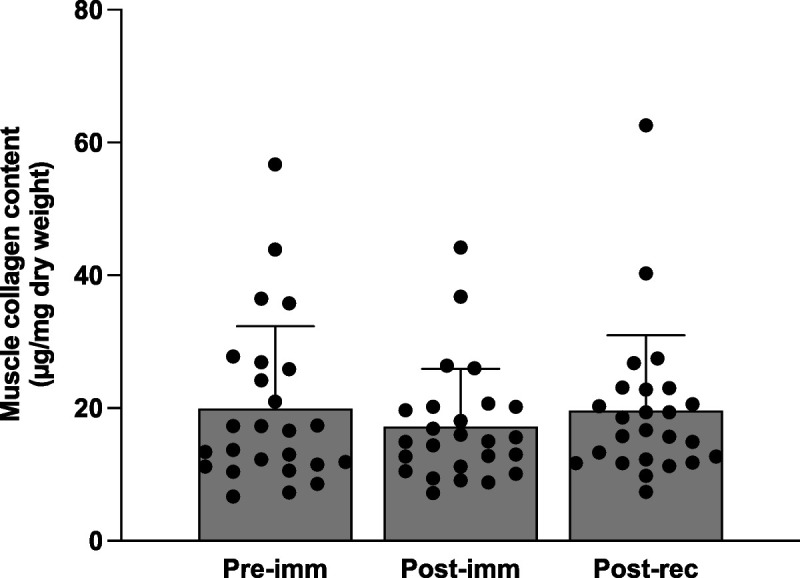
Muscle collagen contents (in micrograms of collagen per milligram of dry weight) in samples collected at baseline (Pre-imm), after 7 d of leg immobilization (Post-imm), and after 14 d of ambulatory recovery (Post-rec). The bars represent means ± SD (*n* = 25), and the dots represent individual values. Data were analyzed using a one-way ANOVA. No significant differences over time were observed in muscle collagen content (*F*_2, 48_ = 0.7, *P* = 0.50).

Data from *n* = 25 subjects were analyzed for changes in muscle collagen contents, because of dropout (*n* = 1) and insufficient muscle tissue (*n* = 4). Muscle collagen contents did not change over time (Fig. 3, *P* = 0.50).

### Connective protein synthesis rates

**FIGURE 4:**
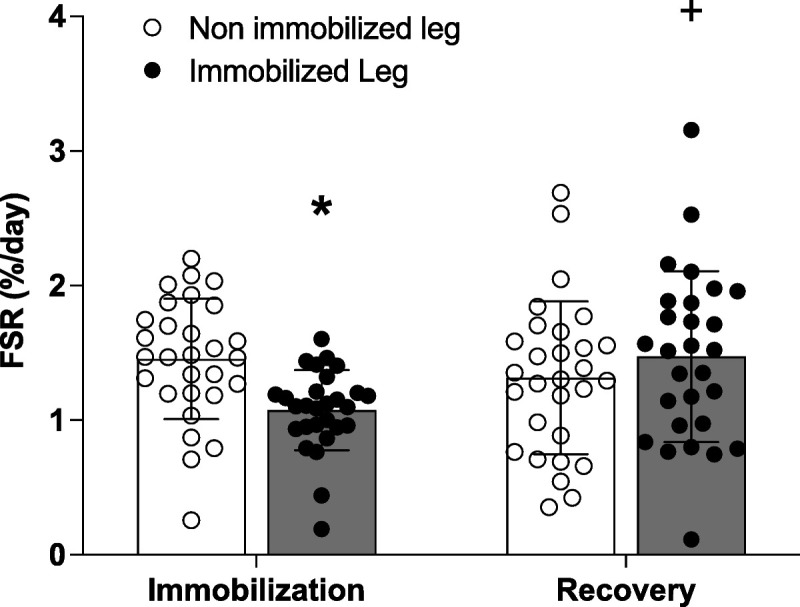
Connective protein FSR (in percent per day) assessed during 7 d of single-leg immobilization and 14 d of ambulatory recovery in the nonimmobilized (white dots) and immobilized legs (black dots). The bars represent means ± SD (*n* = 28), and the dots represent individual values. Data from *n* = 2 subjects (*n* = 1 dropout and *n* = 1 with insufficient muscle tissue) were not included in the present analysis. Data were analyzed using a two-way ANOVA with time and leg as independent variables. A main effect for leg (*F*_1, 27_ = 6.0, *P* = 0.02), but not time (*F*_1, 27_ = 1.4, *P* = 0.25), was observed. A time–leg interaction effect was observed (*F*_1, 27_ = 14.2, *P* < 0.001) *Significantly different from nonimmobilized leg at the same time point (*P* < 0.05). +Significantly different from the immobilized leg during the immobilization period (*P* < 0.05).

Data from *n* = 28 subjects were analyzed for muscle connective protein synthesis rates, because of dropout (*n* = 1) and insufficient muscle tissue (*n* = 1). During immobilization, muscle connective protein synthesis rates were lower in the immobilized (1.07 ± 0.30%·d^−1^) compared with the nonimmobilized (1.48 ± 0.44%·d^−1^; *P* < 0.01) leg (Fig. 4). When compared with the immobilization period, connective protein synthesis rates in the previously immobilized leg increased during subsequent recovery (1.48 ± 0.64%·d^−1^; *P* < 0.01) and did not differ from the nonimmobilized leg during recovery (1.32 ± 0.59%·d^−1^; *P* = 0.23).

### mRNA expression

**FIGURE 5:**
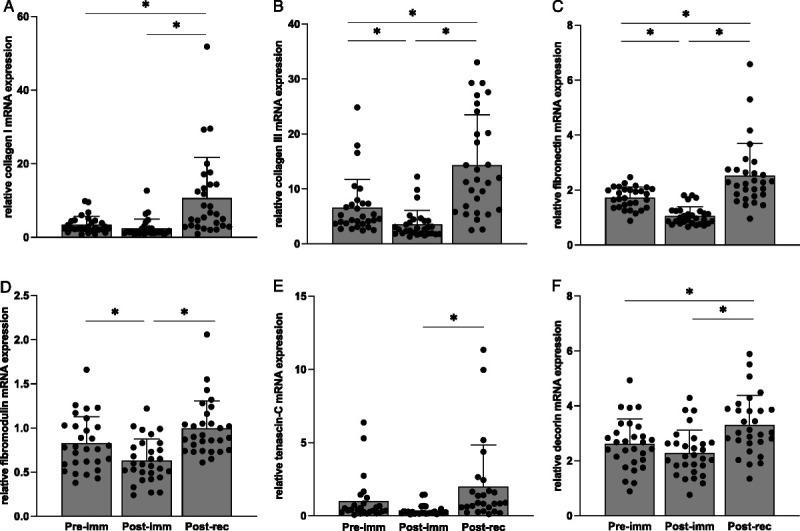
Skeletal muscle mRNA expression of collagen I (A), collagen III (B), fibronectin (C), fibromodulin (D), tenascin-C (E), and decorin (F) before (Pre-imm) and after 7 d of single-legged immobilization (Post-imm) and after 14 d of ambulatory recovery (Post-rec). Data are expressed as means + SD, *n* = 29 for all genes except for tenascin-C (*n* = 26). Main effects for time were observed for collagen I (*F*_1.1, 30.3_ = 12.7, *P* < 0.001), collagen III (*F*_1.4, 38.5_ = 32.6, *P* < 0.001), fibronectin (*F*_1.2, 33.1_ = 29.2, *P* < 0.001), fibromodulin (*F*_1.3, 37.6_ = 18.4, *P* < 0.001), tenascin-C (*F*_1.4, 34.2_ = 5.1, *P* < 0.001), and decorin (*F*_2, 56_ = 12.8, *P* < 0.001). *Significant difference between indicated time points.

After immobilization, collagen III, fibronectin, and fibromodulin mRNA expression were decreased versus preimmobilization (Fig. 5, all *P* < 0.01). After remobilization, collagen I, collagen III, fibronectin, fibromodulin, tenascin-C, and decorin were increased versus postimmobilization (all *P* < 0.01). Postremobilization mRNA expression of collagen I, collagen III, fibronectin, and decorin were also increased versus preimmobilization (Fig. 5, all *P* < 0.01).

### Correlations

Baseline daily step count did not correlate with connective protein synthesis rates during immobilization (*r* = 0.183, *P* = 0.43). The changes in leg extension 1RM did not correlate with the difference in muscle connective protein synthesis rates (immobilized vs nonimmobilized leg; *r* = 0.017, *P* = 0.928) or changes in muscle collagen contents (*r* = −0.327, *P* = 0.095) after immobilization. The changes in leg extension 1RM did not correlate with the difference in muscle connective protein synthesis rates (immobilized vs nonimmobilized leg; *r* = 0.274, *P* = 0.151) or changes in muscle collagen contents (*r* = −0.003, *P* = 0.986) after remobilization.

## DISCUSSION

In the present study, we show that 7 d of lower limb immobilization resulted in a substantial decline in daily muscle connective protein synthesis rates. After immobilization, mRNA expression of key extracellular matrix proteins (i.e., collagen III, fibronectin, and fibromodulin) was decreased when compared with preimmobilization values. Fourteen days of ambulant recovery increased daily muscle connective protein synthesis rates, with concomitant increases in mRNA expression of the key extracellular matrix proteins. Together, these findings provide direct evidence that short-term immobilization compromises muscle extracellular matrix remodeling, which is restored during subsequent ambulant recovery.

Injury and/or illness often requires short periods of inactivity. Periods of inactivity strongly reduce muscle mass and strength (1,2), which can lead to impaired metabolic health and compromised functional capacity (3). Despite its critical role in contractile force transfer, little is known regarding the muscle connective protein synthetic response to inactivity. Here, we subjected male volunteers to 7 d of strict lower limb immobilization and applied deuterium oxide dosing and muscle biopsy sampling to evaluate the impact of short-term immobilization on intramuscular connective protein synthesis rates. As hypothesized, we observed 25% lower connective protein synthesis rates in the immobilized leg during the immobilization period when compared with the nonimmobilized leg (Fig. 4). The decline in connective protein synthesis rates was not associated with baseline daily step count, indicating that habitual physical activity may not have a strong impact on modulating connective protein synthesis rates during immobilization. In agreement, Smeuninx et al. (33) have recently demonstrated that exercise training does not modulate muscle protein synthesis rates during a 5-d period of bed rest in older men. The relative decrease in connective protein synthesis rates aligns closely with the relative decline in myofibrillar and mixed muscle protein synthesis rates reported after short-term immobilization (2,4–6,31,34–36). The similarity in responses to inactivity indicates close coordination between the downregulation of myofibrillar and connective muscle protein fractions that are respectively involved in generating and transmitting contractile force. Our findings extend upon data from our research group (37) and others (38,39) demonstrating a comparable magnitude and duration of myofibrillar and connective protein synthetic responses after a bout of increased physical activity. Overall, we provide the first evidence that a decline in physical activity due to (short-term) immobilization downregulates muscle connective protein synthesis rates in humans.

Despite the decline in muscle connective protein synthesis rates (Fig. 4), we observed no changes in muscle collagen contents after immobilization (Fig. 3). This finding aligns with prior studies in rodents showing no changes in absolute muscle collagen contents after up to 28 d of limb immobilization (20,21,40). Given the relatively short immobilization period, it may be speculated that a decrease in muscle collagen content would be detectable after more prolonged periods of immobilization. However, Haus et al. (41) reported no changes in soleus muscle collagen contents after 35 d of unilateral immobilization in humans. Intriguingly, the same study reported a marked increase in soleus collagen content after 90 d of strict bed rest (41). However, the authors cautioned that the observed increase in collagen contents after bed rest may be due to abnormally low baseline collagen values before bed rest. In fact, a more recent study reported no changes in collagen content within vastus lateralis muscle after the same duration of bed rest (42). Altogether, our data provide support that the decline in muscle connective protein synthesis rates with immobilization does not impact total muscle collagen content.

The decline in muscle connective protein synthesis reflects remodeling responses of extracellular matrix components with specific functional and structural roles and does not need to result in changes in absolute collagen content of muscle tissue. To gain more detailed insight into the complexity of the extracellular matrix remodeling response, we measured gene expression of a variety of key extracellular matrix proteins and glycoproteins. After immobilization, we observed a decline in collagen III, but not collagen I mRNA expression (Fig. 5). Our findings align with two human immobilization studies (43,44) and suggest that immobilization may induce a shift in the ratio of collagen I and III composition in skeletal muscle over time. A shift in the collagen I to III composition has been suggested to alter tissue mechanical properties (40). Specifically, an increase in collagen I to III ratio would result in stiffer tissue, which has been observed in human muscle after ~40 d of immobilization (45), in rodents at the muscle region of the tendon after denervation (46), and in rodent skeletal muscle tissue with aging (47). Furthermore, we observed a decrease in fibronectin and fibromodulin mRNA expression (Fig. 5). Fibromodulin regulates collagen organization (48) and fibronectin facilitates cell-matrix adhesion (49). Heinemeier et al. (50) showed an increase in fibronectin, but not fibromodulin expression in human muscle after a single bout of physical activity. Therefore, fibronectin appears to be sensitive not only to increased mechanical loading (50), but also to unloading as demonstrated here. Overall, the observed pattern of gene regulation would appear to compromise the preferred composition of the extracellular matrix and its binding capability with adjacent muscle fibers.

During the subsequent 14-d remobilization period, daily connective protein synthesis rates increased in the previously immobilized leg when compared with values observed during immobilization and did not differ from the nonimmobilized leg during recovery (Fig. 4). The present values represent average muscle connective protein synthesis rates over the 14-d recovery period. Therefore, we can only speculate as to the time course by which muscle connective protein synthesis rates returned to baseline values after immobilization. Furthermore, as we examined remobilization after a relatively short immobilization period, we can only speculate whether more prolonged immobilization periods would impact the magnitude and/or time course of the connective protein synthetic response during remobilization. Several studies have shown that a single bout of physical activity robustly increases muscle connective protein synthesis rates over the subsequent 4–7 h (14,15,17,19,37,39,51,52). Therefore, we speculate that the reintroduction of ample physical activity stimulates an increase in muscle connective synthesis rates during the initial day(s) of recovery from immobilization, regardless of the length of the immobilization period. Future research characterizing the time course of recovery of muscle connective protein synthesis rates would aid in developing strategies that promote more rapid skeletal muscle recovery from a period of disuse.

Based on our observations that muscle collagen contents did not change after immobilization, it was not surprising to observe no changes in (absolute) collagen contents after remobilization (Fig. 3). In agreement, an earlier rodent study has also demonstrated no changes in absolute muscle collagen contents after 14 d of remobilization after 7 d of limb immobilization (53). We did, however, observe a potent upregulation in the gene expression of all extracellular matrix components that were measured (Fig. 5). The elevations in gene expression after 14 d of returning to normal physical activity indicate muscle connective matrix transcriptional responses that last long into the ambulant recovery period. In support, long-lasting transcriptional responses have also been observed by others reporting an upregulation in mRNA expression of various muscle collagen types for up to 30 d after a single bout of exercise or muscle-damaging activity (50,54,55). Interestingly, we observed that muscle connective matrix components involved in force-transfer (i.e., collagen I, collagen III) and matrix-cell adhesion (i.e., fibronectin) showed even greater mRNA expression after remobilization when compared with preimmobilization values (Fig. 5). Such robust responses in these components may indicate that (re-)strengthening of the muscle matrix and cell adhesion are prioritized in the recovery process.

Plasma markers, such as PINP and CTX-I, are often used as a proxy to evaluate changes in whole body collagen synthesis and breakdown (56). We observed no changes in plasma PINP or CTX-I concentrations after the immobilization and subsequent recovery period (Fig. 2). Our findings directly align with those of Christensen et al. (57), who observed no changes in serum PINP or urinary CTX concentrations after 2 weeks of immobilization and 2 weeks of recovery in healthy men. Our data extend upon these findings and illustrate that changes in connective protein remodeling with immobilization are confined to local tissues (i.e., skeletal muscle) and may not be of sufficient magnitude to impact systemic markers of whole-body collagen remodeling. Although exercise has been shown to increase plasma PINP concentrations (37,58), it seems as though the reintroduction of physical activity after immobilization may not represent a potent enough stimulus to impact whole-body collagen remodeling.

## CONCLUSIONS

In conclusion, 7 d of lower limb immobilization substantially reduces daily muscle connective protein synthesis rates and mRNA expression of collagen III and glycoproteins involved in extracellular matrix organization (fibromodulin) and cell adhesion (fibronectin). Fourteen days of ambulant recovery increases daily muscle connective protein synthesis rates, with robust increases in mRNA expression of collagen I and III and extracellular matrix components. Together, these findings provide direct evidence that short-term immobilization impairs muscle connective matrix remodeling, which is restored during subsequent ambulant recovery.
